# Parents’ perceptions on policies of early childhood care and education programmes in selected states of Malaysia

**DOI:** 10.12688/f1000research.122443.1

**Published:** 2022-08-16

**Authors:** Dandan Tang, Fong Peng Chew, MohdNazri Abdul Rahman, Mogana Dhamotharan

**Affiliations:** 1 Faculty of Education, University of Malaya, Malaysia; 2 Faculty of Education, SEGI University Kota Damansara, Selangor, Malaysia

**Keywords:** Policies, Challenges, Parents’ perceptions, ECCE programs

## Abstract

Background:

The 2030 agenda for sustainable development proposed global equitable quality education and lifelong learning opportunities for all children **. **The quality of early childhood care and education (ECCE) programs helps shape children’s minds, attitudes and behaviors, and has short and long-term effects on a child, a family and a country. In Malaysia, the government has formulated some policies and laws to protect children’s rights. However, ECCE is facing some challenges. The purpose of this study is to investigate parents’ perceptions of the quality of ECCE programs implemented by Malaysian government.

Methods: A mixed method was used to collect data on parents’ perceptions of ECCE policies in selected states in Malaysia. The questionnaires, (P1/POL) from the research project “Development of a Comprehensive and Integrated Model of Quality Malaysian ECCE”, were distributed among 629 respondents who have a child in a preschool, and 22 participants were randomly selected to take part in five focus group interviews

Results: The key findings of the study revealed 68% parents were not familiar with ECCE Malaysian government policy, however 84.3% stressed it is important for the government to educate them about ECCE. Thus findings indicated that the majority of parents lack awareness of the ECCE policies and quality of early childhood care and education programs related to the policies remain the issue. While interviewing the focus group ,most of them were not aware of ECCE and pointed out parents are stressing children’s academic learning in particular preschools.

Conclusions: It is concluded that parents’ awareness regarding the ECCE program must be part of the policies and needs to improve. It is recommended that the government of Malaysia should supply more information on ECCE policies to parents and focus on policy implementation. Moreover, the quality of ECCE programs should be improved based on the parents’ perceptions.

## Introduction

The definition of early childhood care and education (ECCE) is the “holistic development of a child’s social, emotional, cognitive and physical needs in order to build a solid and broad foundation for lifelong learning and wellbeing.” ( UNESCO, 2005). The 2030 Agenda for Sustainable Development meeting proposed that all countries should ensure inclusive and equitable quality education and facilitate lifelong learning opportunities for all ( UNESCO, 2015, p.21). In addition, the State of the World’s Children reported that efforts to protect and expand an individual’s right to education should begin in early childhood. Moreover, it is the right of every child to receive a quality education ( UNICEF, 2016). ECCE programs are places that enable children to shape their minds, attitudes and often behaviors ( Ortiz, 2015).

On the other hand, parents’ engagement and education in early childhood is the foundation of all the education and has significant implications for a child’s well-being and success in later life ( Alameda-Lawson, 2014; Barnes, *et al.*, 2016). The influence of parents on children's growth process has basic, enlightening, subtle and natural characteristics ( Goodall, 2017). Early childhood programs and parents share the responsibility to make contributions to the partnership for the benefit of the child. The relationship between parent and school is evolving to pay attention to partnerships and two-way communication (Epstein, 2010; Barnes *et al.*, 2016). Therefore, parents have important implications for the ongoing process of evaluating, monitoring and improving the quality of ECCE programs.

Moreover, early childhood care and education have the policies and procedures on a whole range of practice issues ( Fitzgerald and Kay, 2016, p.6). Fitzgerald and Kay (2016) stress the importance of early years policy for practitioners, children, and parents. Early years policies are aimed at children and families, they impact practitioners significantly to have appropriate qualifications and skills to work with children. Moreover, the policy changes and implementations have implications for children’s diverse developmental needs. Parents can make contributions to the work of providers. Leonidas Kyriakides (2005) found that the implementation of an active partnership policy can provide parents with the opportunity to be involved in the development of school policy. Parents have an active response concerning the impact of school policy. It is suggested that working effectively with parents demands policies that meet the parents’ requirements ( Fitzgerald and Kay, 2016, p.138).

Currently, countries around the world have recognized the importance of early childhood development and its impacts on the countries’ long-term economic and social developments ( Hardin *et al.*, 2017; Foong *et al.*, 2018). The quality of early childhood education has been improved across the world since the Millennium Development Goals and Education for All targets were proclaimed ( UNESCO, 2014; Engdahl, 2015; Shaeffer, 2016). Considerable progress has been achieved in promoting the well-being and development of young children, especially in European countries and the United States, where they account for the best practices of their early childhood education. The improved quality and equality of early childhood education in European countries and the United States have put much focus on the development and establishment of effective policies and practices ( Hägglund, & Johansson, 2014; Shaeffer, 2016).

According to UNESCO (2021) globally early child education enrolment rate in school has increased by 27% from 34% in 2001 to 61% in 2019. Although there is significant progress of early child education enrollment in 2019, globally however about 175 million children 3-6 years old are out of the school and have not received quality education.

Abudu and Fuseini (2013) have also stressed that the increasing income gap has slowly enlarged the gap in early childhood education (ECE) quality in Asian countries, especially the quality of early childhood education between rural and urban areas. It is necessary for the government to establish and set effective policies to protect children’s rights to acquire high-quality early childhood care and education ( Shaeffer, 2016; Samuelsson & Park, 2017; Shakeel, & Aslam, 2019).

In Malaysia, various approaches have been taken by the government to ensure and enhance the high quality of ECCE. ECCE is treated as a precondition to quality education as it is the primary factor for developing and improving young children’s knowledge, social skills, and attitudes toward life during one’s early childhood years ( Myers, 2004; *Eleventh Plan Malaysia 2016-2020: Anchoring Growth on People,* 2015). ECCE consists of preschools for children aged 4 to 6 years old, and childcare centers for younger children aged 0 to 4 years old. The enrollment in preschool has increased from 46.24% in 2000 to 83.2% in 2013( McCool, 2013) In 2016, there were 200,684 pupils enrolled in preschool programs ( *Malaysia National Education for All Review Report,* 2015; Samuel & Tee, 2017). However, the Education Blueprint of Malaysia acknowledges that serious problems remain with the quality of education and the investment in education is not as high as expected ( *Malaysia Education Blueprint 2013-2025*, 2013). In addition, parents’ awareness of the positive impact of preschool and their expectations for preschool programs has increased in Malaysia ( Siti Naziera, Jati Kasuma, & Irwan Shahrinaz, 2017; Tang *et al.*, 2021).

Parental perspectives related to the quality of early childhood care and education programs are critical because parental perceptions reflect parental involvement and directly influence the quality of education ( Ortiz, 2015). However, parents are rarely included as participants in research in the field of early education ( Vuorinen, 2018). According to the *Malaysia National Education for All Review Report* (2015), the majority of parents were not satisfied with the quality of education in preschool and they argue that ECCE is mostly like a playground for their children.

### The history of ECCE in Malaysia

The history of early childhood care and education started in the 1950s. From 1950 to 1960, kindergartens in Malaysia were opened by socialist Christians as part of church activities. Almost all kindergartens were owned by individual or private agencies in town areas. The fees were expensive to serve the children who were from rich families. Around 1969, the Asia Foundation contributed financial assistance to the Worker Society of Malaysia to develop a model of kindergarten called Taman Didikan Kanak-Kanak (TADIKA) in the Malay language similar to a project in the United States ( Hutchins, 1995) In 1970, the launching of the New Economic Policy which focused on the eradication of illiteracy by the Department of Community Development (KEMAS) was established. The department of Neighbourhood and National Unity and the Ministry of Federal Territory made a decision to establish Taman Didikan Kanak-Kanak (TADIKA) or Taman Bimbingan Kanak-Kanak (TABIKA) for children aged 4 to 6 years. In 1972, the Ministry of Education enacted a special parliamentary act for early childhood education called Education Act 1961 ( Ministry of Education, 2020).

From 1972 to 1976, Maktab Perguruan Ilmu Khas (MPIK) (Special Education Teaching College) offered a course to train teachers in early childhood education. After that, the same programs were developed by the teaching colleges and government universities in Malaysia. In 1984, the Children Centre Act was established and in 1986 the Preschool Teachers Guide Book was published. This book was published for the purpose of enhancing the skills and creativity of preschool teachers.

From 1990, government departments were actively developing early childhood education, and the number of early childhood education institutions continued to increase, broadening the education of children. In 1993, the Malaysian Preschool Education Guidelines were introduced and adopted by preschools under the Ministry of Education ( Heng, 2008; Suseela Malakolunthu, & Nagappan. Rengasamy, 2012). According to the Malaysian Education Blueprint (2012-2025), clear targets were set in terms of quality early childhood education. One of the first stage targets is that the enrollment rate reaches 92% in preschools. All the preschools must be registered and abide by the regulations of the Ministry of Education. The National Early Childcare & Education Curriculum (PERMATA, 0-4 years) and National Preschool Curriculum Standards (KSPK, 4-6 years) are the main references for early childhood curriculum in a preschool. In accordance with the guidelines of the preschool education curriculum, teachers inspire children's intelligence and creativity by playing games and playing with teaching. The education bureaus in the states supervise whether the kindergartens comply with educational guidelines (UPSI National Education Museum, Tanjung Malim, Malaysia; Shaeffer, 2016).

### Policies of ECCE in Malaysia

The Malaysian government has put forth various laws and guidelines in the field of ECCE. There are educational policies and practices to address cultural diversity in Malaysia including issues and challenges ( Malakolunthu, Rengasamy, 2012; Hooi San Phoon *et al.*,2013). The Education Development Master Plan 2001-2010, the Education Blueprint 2006-2010, and the Education Blueprint 2013-2015 have become the main guidelines for the education sector and securing funding for this sector. The 1984 Child Care Act, amended in 2007, refers to all early childhood care and education programmes for children under the age of 4 ( Child Care Centre Act 1984, 1984). Convention on the Rights of the Child 1989 and Child Act 2001 (amendment in 2016) emphasized a child’s right to gain an education. The Education Act (1996) has included preschool education as part of the national school system. The National Education Act and Childcare Centre Regulation 1972 (revised draft 2012) formally integrated pre-primary education into the education system. In addition, some guiding policies which are related to early education were formulated such as Malaysian Education Blueprint (2012-2025), Eleventh Malaysian Plan (2016-2020), Country Health Plan 2015, and National Early Childcare and Education Policy 2008. However, the implementation of these policies is less than satisfactory ( Ng, 2010; Hooi San Phoon *et al.*, 2013).

### Policies of ECCE programmes in Malaysia

According to the policy, ECCE in Malaysia is broadly divided into two age groups including 0-4 years old and 4-6 years old ( *Early Childhood Care and Education Policy Implementation Review*, 2008). Ministry of Women, Family and Community Development takes over responsibility for child care and coordinates national programs on the growth and development of children ages zero to four years old in childcare centers (also known as Taman Asuhan Kanak-Kanak). Preschool education (also known as Taman Didikan Kanak-Kanak) for the children who are four to six years old comes under three ministries, namely the Ministry of Education, the Ministry of Rural and Regional Development, as well as the National Unity Department. Moreover, to ensure quality teaching, the National Preschool Curriculum Standards and National Early Childcare and Education Curriculum used in preschools are standardised to ensure that all pupils are able to become literate and calculate before beginning formal education at the primary level ( *Mid-term Review Ninth Malaysia Plan 2006-2010*, 2010).

A significant change was made in preschool education in Malaysia in 2001. “The Council of Ministers gave its approval for the expansion of Preschool Programmes in education and also articulated in the National Preschool Curriculum that learning and teaching would be enacted in a more holistic, interesting and more orderly way” (Abdul Halim Masnan, 2010, p.3). To guarantee children’s health and safety, National Child Policy and Action Plan, National Family Policy and Action Plan, National Nutrition Policy as well as Child Health Services Policy were carried out. The National Preschool Quality Standard (SKPK) and National Quality Assurance System (PERMATA Q) are important policies implemented for the assessment of quality standards for preschools in Malaysia ( Raman & Sua, 2010; Zalizan, Ali Manisah Mohd, 2014; Cheong, Hill & Leong, 2016).

### Quality ECCE programmes and parents’ perspectives on ECCE Policies

Lassalle and Nektarios (2018) stated that several conditions contribute to the quality ECCE programs such as the learning environment, professional teachers and caregivers, the relationships among families and teachers as well as communities. Moreover, ECCE policy implementation is important in preschools. Bennett and Neuman (2004) argued that one of the main means of ensuring high-quality education for children is to formulate national education frameworks and policies and provide teachers and parents with intensive in-service training so that they can understand and comply with relevant laws and regulations. It is necessary to pay attention to parents’ perspectives and the interaction between parents and teachers. However, parents are rarely included as participants in research ( Vuorinen, 2018) and therefore parents are not aware of the policies. Lack of correct parenting perspectives involving cultural, ethnic or personal values, or lack of interest in acquiring early childhood knowledge that could benefits parents to understand children rights for the education, can result in the the short and long term lack of educational awarness. ( Vargas-barón, 2016). Some ECCE policies are brief “policy statements”, formally establishing the country’s intention on young child development. Inadequate attention of parents’ and children’s rights organzations is the reason ECCE polices are not sucessfully implemented ( Vargas-barón, 2016). Haddad (2002) concluded an overview of the current situation and recent changes in ECCE policy in developed and developing countries and reported that new government policies have brought about a wide range of reforms addressing the family influence on children’s development in a developed country.

In Malaysia, many comprehensive policies are in place. However, the implementation of the policies is less than satisfactory ( Ng, 2010). Although inclusive education was implemented at the policy level for more than ten years, Malaysia is far from reaching the goal of providing a responsive education path for every child ( Zalizan & Manisah Mohd Ali, 2014, p.997). On the other hand, parents lack “political will” which is linked to political interests; cultural, national, or personal values, and they are concerned about the information and resources of early education ( Kunagaratnam, & Loh, 2010; Vargas-barón, 2016). This paper focuses on the perspectives of parents on the policy implementation of ECCE programs.

### Aims of the study and research design

The general aim of the study is to investigate parents’ perspectives of quality ECCE programs related to policies. The study aims to answer the following questions:

1. Do parents know the policies of early childhood care and education?
2. In what way do parents know the policies of ECCE?
3. What are the main challenges of quality early childhood care and education programs related to policies from parents’ perspectives?

An explanatory mixed research design was employed in this study to investigate parents’ perceptions on the policy of the ECCE, and the main challenges faced in the implementation of ECCE. The first intent of the research was to collect and analyze quantitative data to find out parents’ views of existing ECCE policies. For this purpose, a questionnaire was distributed to parents in the selected states of Malaysia and provided the general outcomes of the study. Then the researcher engaged in a qualitative phase by doing focus group interviews to help explain the quantitative research results. The triangulation of the data helps to capture the overall picture of ECCE policy implementation of quality ECCE programs in Malaysia and evaluate the policy implementation in preschools based on parents’ perceptions which is beneficial for preschools to improve the quality of education and promote the further implementation of the ECCE policy in Malaysia.

## Methods

### Ethics and consent

This study examined government policies for the early childcare education in Malaysia under the national research project titled “Development of a Comprehensive and Integrated Model of Quality Malaysian ECCE”. This study is approved by the Institutional Review Board from the SEGI University with the approval number: SEGi/LRGS/2015-0024-106-04 on 6 September 2017. Informed written (questionnaires) and oral (focus groups) consent was taken from participants who wished to take part in this study.

30 participants were approached for the focus group interviews. 22 gave consent to participate and 8 refused and were not included further. The COREQ guidelines were followed in reporting this research.

### Reflexivity

The researchers are familiar with the study location and did not face any problems e reaching out to the participants. Researchers first established a good rapport with participants and explained the purpose of the study. This way we developed the trust of the participants in the researchers. The researchers and participants did not have a relationship prior to the study.

Three authors hold PhDs, and one author is in the final year of their PhD. All authors have prior experience with ECCE research and practice.

### Participants

The participants are parents who have children in an ECCE programme in the selected states of Malaysia: Selangor, Kuala Lumpur, Pulau Pinang, Kedah, Perak, Kelantan, Terengganu, Johor, Negeri Sembilan, Pahang, Sarawak, and Sabah. Tadika and Taska schools for the ECCE in the urban and rural areas among the 12 states were selected using a systematic sampling method to cover maximum number of parents. While picking up or dropping off their children to the schools, parents were approached by the research team to take part in the study. The study purpose was explained to them and questionnaires distributed in-person to those who consented to take part.

### Questionnaires

The questionnaire (P1/POL for parents) from the national research project entitled “Development of a Comprehensive and Integrated Model of Quality Malaysian ECCE” which is used to examine current government policies, regulations and legislation pertaining to Malaysian ECCE in the context of integration between policy and practice was used in this study. The questionnaire consisted of demographics, parents’ views of policy including nine items, and parents’ views of ECCE programs including 62 items. The validity and reliability of the questionnaire were measured and considered excellent (Cronbach’s alpha= 0.932). A total of 629 questionnaires were distributed to participants and 543 usable valid questionnaires were used in this research study. Statistical Package for the Social Sciences (SPSS.20) was used to conduct the analysis of the quantitative data to answer the research questions “do parents know the policies of early childhood care and education, and in what way do parents know the policies of ECCE”. Table 1 shows the demographics of the respondents.

**Table 1.** Participants demographic information (N=543; SPM: Malaysian Certificate of Education).

Items		Frequency	Percentage (%)
Qualification	SPM	247	45.50
	Diploma	86	15.80
	Degree	184	33.90
	Master	19	3.50
	PhD	07	1.28
Gender	Male	216	39.8
	Female	330	60.2
Age	Below 20 years	13	2.40
	21-30 years	155	28.55
	31-40 years	271	49.90
	Above 41 years	104	19.15
Race	Malay	174	32.00
	Chines	269	49.50
	Indians	73	13.45
	Others	27	5.0
States Residence	Kedah	4	0.73
	Kelantan	13	2.40
	Selangor	289	53.22
	Pahang	09	1.65
	Pulau Pinang	30	5.5
	Perak	7	1.28
	Negri Sembilian	9	1.65
	Terengganu	5	0.92
	Johar	15	2.75
	Sabah	24	4.40
	Kuala Lumpur	108	19.90
	Sarawak	28	5.15
Early child care school type	Taska	31	5.70
	Tadika	512	94.30

### Focus groups

30 participants were randomly selected to take part in the focus group interviews. 8 refused to participate without giving a reason and were not included further. A total of 22 participants took part in focus group interviews. The interviews were conducted in a private meeting room in a nearby school. There was no one present beside the participants and researchers. Field notes were taken in all interviews, and the groups who consented to it were audio recorded (groups 1-3). Interviews lasted approximately 20 minutes.

There were five participants from Selangor, six participants from Kuala Lumpur, six participants from Sarawak, two participants from Melaka, two participants from Pahang, and one participant from Sabah, and they were in five interview groups. The audio recordings were transcribed and the transcriptions and field notes were analyzed by using Nvivo V.10 software. The portion of data was coded during the first and second cycle coding process. Codes were established for key concepts related to the research and based on what outcomes from the parents’ perceptions. The themes were derived from the codings, categorization and analytic reflection based on parents’ perceptions on the policies and challenges of quality ECCE programmes.

## Results

### Quantitative data demographic analysis

Table 1 indicates the demographic information of the participants and shows most of the respondents (N=330) are female. Half (N=271) of the respondents are aged 31-40 years old, and the highest qualifications are SPM (Malaysian Certificate of Education; N=247) followed by a degree (N=184). Half of the respondents are Chines (N=269) followed by Malay race (N=174), and among 12 states of the Malaysia most of the respondents are from the Selangor and Kuala Lumpur. In Malaysia, two major early child care education programs approved by the government are called Taska and Tidka; Taska mainly deals with early child care for ages 0-4 years and a nursing program, while Tidka accepts children age 4. In this study most of the parents were registered with Tidka schools.

To investigate whether parents know the policies of ECCE, the frequency and percent of policy documents were analyzed. For the answers to the item “It is important for parents to know the policy documents”, 457 (84.2%) chose “Yes” and 86 (15.8%) chose “No”. It is obviouhs that the majority of parents thought they should know the policy about early education.

Table 2 presents the results of item “In your search for a quality ECCE setting/center for your child, which of the following policy documents have you referred or consulted?”. 21% (n=116) of parents refer to policy documents *Malaysian Education Blueprint (2012-2025)*17) and *National Preschool Curriculum Standards (KSPK)* (n=116, 21.4%). 20% of parents would like to refer to the *Education Act 1996 (Act 550)* (n=110, 20.3%) and *National Preschool Quality Standard (SKPK)* (n=109, 20.1%).

**Table 2.** Policy documents parents have referred to or consulted.

Policy document	Frequency	Percent (%)
**Guiding policies**		
1) Malaysian Education Blueprint (2012-2025)	116	21.4
2) Eleventh Malaysian Plan RMK-11 (2016-2020)	46	8.5
3) Country Health Plan 2015	35	6.4
4) National Blue Ocean Strategy	25	4.6
5) National Education Policy 2012	70	12.9
6) National Early Childcare & Education Policy 2008	16	2.9
**Registration**		
7) The Childcare Centre Act 1984 (revised 2012) (Act 308)	99	18.2
8) Education Act 1996 (Act 550)	110	20.3
9) Childcare Centre Regulation 1972 (revised draft 2012)	82	15.1
10) Education (Special Education) Regulations 2013	11	2.0
11) Special Education Students Practice Code 2015	6	1.1
12) National Land Code 1965 (Act 56)	23	4.2
13) Town and Country Planning Act 1976 (Act 172)	27	5.0
14) Local Government Act 1976 (Act 171)	26	4.8
15) Uniform Building By-Laws 1984	19	3.5
16) Workers’ Minimum Standards Housing Amenities Act 1990	22	4.1
**Curriculum**		
17) National Preschool Curriculum Standards (KSPK)	116	21.4
18) National Early Childcare & Education Curriculum	80	14.7
**Rights of the Child**		
19) Convention on the Rights of the Child 1989	17	3.1
20) Sexual Offences against Children Act 2017	60	11
21) Child Act 2001 (Act 611) Child (Amendment) Act 2016	21	3.9
22) Aboriginal People’s Act 1954 (Act 134)	3	0.6
23) Persons with Disabilities Act 2008 (Act 685)	29	5.3
**Health and Safety**		
24) National Child Policy and Action Plan	63	11.6
25) National Child Protection Policy and Action Plan	76	14
26) National Family Policy and Action Plan	50	9.2
27) National Nutrition Policy	51	9.4
28) Child Health Services Policy	65	12
29) Food Act 1983 and Food Regulations 1985	45	8.3
**Assessment of Quality Standards**		
30) National Preschool Quality Standard (SKPK)	109	20.1
31) National Quality Assurance System (PERMATA) Q)	40	7.4

Like other policy documents, a minority of parents refer to them. The number of respondents (parents) who refer to the policy documents was below 30%. That means more than 70% of parents do not refer to the policy documents about ECCE in Malaysia.

Table 3 shows the analysis results of the item “In your view, which of these policy documents are important for parents to know?”. 33% (n=177) of parents reported that the policy about the curriculum of “ *National Preschool Curriculum Standards (KSPK)*” (n=177, 32.6%) is the most important. Parents also cared more about *Childcare Centre Regulation 1972 (revised draft 2012)* (n=172, 31.7%), the *Malaysian Education Blueprint (2012-2025)* (n=170, 31.3%), *the Childcare Centre Act 1984 (revised 2012)* (n=167, 30.8%) *, Education Act 1996 (Act 550)* (n=145, 26.7%) as well as *National Preschool Quality Standard (SKPK)* (n=142, 26.2%) and *Child Health Services Policy* (n=140, 25.8%). This indicates that parents consider the importance of policies that are directly related to their children’s ECCE center.

**Table 3.** Parents’ views on the importance of policy.

Policy document	Frequency	Percent (%)
**Guiding policies**		
1) Malaysian Education Blueprint (2012-2025)	170	31.3
2) Eleventh Malaysian Plan RMK-11 (2016-2020)	78	14.4
3) Country Health Plan 2015	67	12.3
4) National Blue Ocean Strategy	39	7.2
5) National Education Policy 2012	120	22.1
6) National Early Childcare & Education Policy 2008	35	6.4
**Registration**		
7) The Childcare Centre Act 1984 (revised 2012) (Act 308)	167	30.8
8) Education Act 1996 (Act 550)	145	26.7
9) Childcare Centre Regulation 1972 (revised draft 2012)	172	31.7
10) Education (Special Education) Regulations 2013	22	4.1
11) Special Education Students Practice Code 2015	16	2.9
12) National Land Code 1965 (Act 56)	19	3.5
13) Town and Country Planning Act 1976 (Act 172)	36	6.6
14) Local Government Act 1976 (Act 171)	46	8.5
15) Uniform Building By-Laws 1984	35	6.4
16) Workers’ Minimum Standards Housing Amenities Act 1990	36	6.6
**Curriculum**		
17) National Preschool Curriculum Standards (KSPK)	177	32.6
18) National Early Childcare & Education Curriculum	92	16.9
**Rights of the Child**		
19) Convention on the Rights of the Child 1989	27	5.0
20) Sexual Offences against Children Act 2017	127	13.4
21) Child Act 2001 (Act 611) Child (Amendment) Act 2016	24	4.4
22) Aboriginal People’s Act 1954 (Act 134)	7	1.3
23) Persons with Disabilities Act 2008 (Act 685)	40	7.4
**Health and Safety**		
24) National Child Policy and Action Plan	102	18.8
25) National Child Protection Policy and Action Plan	110	20.3
26) National Family Policy and Action Plan	72	13.3
27) National Nutrition Policy	92	16.9
28) Child Health Services Policy	140	25.8
29) Food Act 1983 and Food Regulations 1985	84	15.5
**Assessment of Quality Standards**		
30) National Preschool Quality Standard (SKPK)	142	26.2
31) National Quality Assurance System (PERMATA) Q)	52	9.6

For the answers to the item *‘Information about the policy documents can be disseminated to parents through’,* most of the parents (N=392) stated school can disseminate policy documents for them. However, N=111 parents stated policy documents can be propagated through the channel of social medial and N=40 parents chose option for community disseminating, hospital conducting awareness program.

40% (n=249) of parents reported that policy documents can be propagated through the channel of social media. 16% (n=16) of parents chose others such as community disseminating, hospital conducting propaganda and government.

Interview: qualitative data analysis

Demographic information of the parents involved in the interview process are given in Table 4 below. A total of 22 respondents volunteered for the interview program.

**Table 4.** Demographics of respondents in focus group interviews (Malaysia, author source data).

Group	Age	Gender	State	Occupation	Enrollment time	Average number in child’s class	Education fee per month
P1.1	32	Female	Selangor	Housewife	3 years	20	RM250
P1.2	35	Female	Selangor	Housewife	2 years	10	RM250
P1.3	29	Female	Selangor	Housewife	2 years	10	RM250
P1.4	40	Male	Selangor	Housewife	4 years	15	RM250
P1.5	53	Female	Sabah	Educator	2 years	18	RM250
P1.6	39	Female	Selangor	Housewife	6 months	20	RM20
P2.1	36	Female	Melaka	Housewife	2 years	14	RM15
P2.2	45	Female	Melaka	Housewife	2 years	10	RM15
P3.1	30	Female	KL	Housewife	1.5 years	7	RM200
P3.2	48	Female	KL	Housewife	1year	6	RM200
P3.3	43	Female	KL	Housewife	3 years	6	RM240
P3.4	38	Female	KL	Housewife	3 years	6	RM240
P3.5	32	Male	KL	Seller	6 months	7	RM240
P3.6	40	Female	KL	housewife			RM300
P4.1	37	Female	Pahang	Housewife	2 years	5	RM170
P4.2	27	Female	Pahang	Housewife	2 years	24	RM170
P5.1	25	Female	Sarawak	Housewife	1year	20	RM20
P5.2	37	Male	Sarawak	worker	2 years	18	RM20
P5.3	28	Female	Sarawak	Housewife	2 years	20	RM20
P5.4	35	Female	Sarawak	Tailor	1 year	17	RM20
P5.5	38	Female	Sarawak	Housewife	2 years	19	RM20
P5.6	39	Female	Sarawak	Housewife	2 years	16	RM20

To investigate in-depth information on parents’ perceptions of ECCE policy in Malaysia, the researchers conducted focus group interview with 22 parents. Three themes regarding parents’ perceptions on ECCE policy were obtained from qualitative data, and the results are presented in Table 5.

**Table 5.** Three themes regarding parents’ perceptions on ECCE policy.

Codes	Author sources	Categories	Themes
ECCE policy is important	18		
Not familiar with ECCE policy	15	Lack ECCE policy awareness	Policy awareness
Unknown ECCE policy	12	Know little ECCE policy	
Centers are registered	16	Good practice	
Good environment in urban areas			
Health and safety	12		
Teacher and principals training	4	Expectations	Policy implementation
Quality ECCE programs	6		
Poor hygiene in rural areas	4	Issues	
Not registered, registration not renewed.	5		
Lack assessment by welfare department	3		
disparity between rich and poor areas	5		
Cultural diversity	5	Quality standard and assessment of preschool	Challenges of policy implementation
Discrimination happened	3		
Nobody checks the quality of centers	7		
Stress academic learning	4	Parental involvement	

Themes are policy awareness, policy implementation, and challenges of policy implementation. Two categories are under the theme of policy awareness: lack ECCE policy awareness and knowing little ECCE policy. During the focus group interviews, when the interviewer asked the questions ‘what are some of the policies that you are familiar with?’, 15 (68%) parents stated that they were not familiar with the policies of ECCE. Moreover, when the interviewer asked that ‘ *how did you know about the policy and legislative documents*?’ 12 parents (54%) reported they do not know the policy and legislative documents.

“Something [inaudible segment] but not I don’t know how they (policies) apply this in education.” (P1.4)

“We don’t know the about this policy. We heard about this is a strategy.” (P3.2)

“Err I don’t know about locally la because I also hardly get the opportunity to know err most of the time …” (P5.5)

(Focus group interview)

For the theme of policy implementation, two categories including good practice and expectations were obtained. 16 parents have shown satisfaction on the early child care education center registration and reported good environment in urban areas compared to rural location, moreover, they reported that centers provide healthy food while schooling and ECCE centers are more safe for the children in urban areas because of the policy implementation in ECCE. Moreover, parents expected the quality of teachers to be improved. Some parents pointed out that there were teachers screaming in a particular preschool. Parents thought teachers should be trained to be able to go beyond teaching children through books but also to understand the children who have issues such autism or dyslexia. Parents described that teachers and principals need to be sent for training. A good teacher must acquire some knowledge about psychology. If the teachers understand psychology, they can get the children out of a bad situation. It is better for children to learn how to cope with life problems from teachers. The lessons need to be related to real experiences and teachers should explore suitable teaching methods based on parents’ perceptions. At the same time, parents thought the principals and teachers need to stay relevant, they have to upgrade their facilities constantly and also their own qualifications.

“Some teachers they scream, even in kindergartens in preschools. Even the quality of the teachers should be improved.” (P5.1)

“I think that’s why teachers need to be sent out for training coz I understand now a lot of children also have issues like they get autistic and dyslexia like maybe 10% 20% children … I think teachers have to be trained to be able to go beyond teaching them through books but have to understand the child. You know teaching them how to cope with life measures, I don’t know. You know all sorts of things go beyond that because teachers are the only person who can go up from the situation so the teacher must be quick enough to notice them and to help them at this age. Do you get what I mean ahh? (P3.6)

“So you know teacher should know a bit about psychology, coz it’s very important as they don’t get to, they have to get out, and school is the place where they have to explore, and the teacher is the one who should be sharp enough that they are able to get the children out of that situation.” (P1.2)

“I think the principal and the teacher need to stay relevant, they have to upgrade their facility constantly and also their own qualification. Maybe you are teaching only using paper, but now need to use a computer and videos for teaching but now you may want to explore with other materials. Lessons need to be very relevant to today’s world as children are very clever nowadays.” (P2.2)

The theme of challenges of policy implementation combines three categories: issues, quality standard and assessment of preschool, and parental involvement. When parents talked about the issues raised by centers, they expressed some views on developing quality ECCE programs. They stressed the importance of children’s health and safety. Based on their opinions, centers should provide children with nutrition and a balanced diet. However, parents lamented that there is a poor hygienic environment, particularly in some preschools and no clear action has been taken by the welfare department.

“There’re more erm mosquito. [inaudible segment] they can’t really take care of their hygiene. Sometimes, other than no. I think I will always complain of the mosquito bringing in all that …” (P5.3)

“It also depends on the place right and who runs the place … some teachers in preschool think that the setting in their preschools is fine. The children have safeguarded regards to hygiene. However, we do not think so. It is common if anything falls on the floor. But for our children, we are so particular about. If children’s bottle falls on the ground, they (the teacher or caregiver) just pick it up and brush it and put it in the children’s month. We think it is serious not good for children.” (P4.2)

Moreover, six parents emphasized that the quality of kindergartens in rural areas was poor and some preschools did not register at all or registrations were not renewed. Five parents pointed the disparity in preschools between rich and poor areas.

“Urban schools are still ok, but I am very worried about, concerned about rural. Even in, within KL there are so many other schools like she said “dirty, unsafe … (cross talk).” (P3.2)

“But those are government schools, but not all lah, there was one in PJ that I went to, WOW! Its posh, really cool, air conditioned all over, beautiful! Compared to the other one I went to, there is a vast difference, it’s like you walk out in Singapore and then back to Johor.” (P1.6)

“But I have seen so many childcare around my house you know, those are run down, dirty, I don’t know how to help those, those are super dirty, especially the unregistered child cares so the government needs to work towards that.” (P5.3)

In addition, to the quality standard and assessment of preschool, five parents mentioned that cultural diversity remains an issue. Three parents stated that discrimination still happens in particular preschools. Furthermore, seven parents complained that nobody checks the quality of the center.

“Says she was ahh being discriminated upon because of the color. She got a very dark- quite a dark complexion and ahh all-all the other children keep teasing her and all that and all that… So my wife ask her whether you talk to the teacher, she said she doesn’t tell the teacher, afraid that ahh that the children will [inaudible segment] on this type of thing.” (P5.2)

“Actually we have the Malaysian standard which is very good already, if you go through the whole document, all the kindergartens do very good things (inaudible segment), it’s just that nobody check what should I follow and I think that is the issue.” (P3.5)

On the other hand, four parents commented that they are putting too much emphasis on academic success for children in their early years’ education. They described that they hope their children could learn more and have good preparation in kindergarten for transitioning to primary school. Some parents suggested that preschools should slowly improve their academic skills by using suitable approaches.

“I guess Malaysia parents will give more importance to academic based curriculum instead of play based because they don’t believe that children learn just through play. Now you see when they go to school they care about the skills of writing and reading. However, it needs to consider when children study in a primary school. Some parents often ask teachers why they are playing all the time. So it’s different.” (P3.1)

“What I heard even the year 1 and year 2 schools, government school is pretty much … umm academic oriented so I think we should ummm slow down specially at the kindergarten level. I mean you can have academic but probably slow the approach and not too much classroom oriented.” (P3.6)

## Discussions

Based on the findings, 84.2% of parents agree on the importance of knowing ECCE policy documents. However, the fact is that 70% of parents did not refer to the ECCE policy documents because 68% of parents were not familiar with ECCE policy documents. This revealed that some parents lacked awareness of the ECCE policies. Some parents even complained that they knew nothing about the policies of early childhood care and education because they do not have the opportunities to learn. This indicates that the policy implementation should be enhanced by the Malaysian government which should supply more information of ECCE policy to parents and follow the *National Child Policy and Action Plan, National Family Policy and Action Plan, National Nutrition Policy* as well as *Child Health Services Policy* to guarantee children’s health and safety. Abide *the National Preschool Quality Standard (SKPK) and National Quality Assurance System (PERMATA Q)* to do the assessment of quality standards of preschools ( Abdul Halim, 2010; Raman & Sua, 2010; Zalizan, Ali Manisah Mohd, 2014; Cheong, Hill & Leong, 2016).

Findings show parents focus on the policies which directly related to children’s ECCE center. This is similar to the study of Nagasangari Kunagaratnam and Loh (2010) who found that parents emphasize the importance of the information and resources of the preschool. According to findings, 72.2% (n=392) of parents hold the view that preschool can provide them with information about ECCE policy documents. A total of 20% (n=111) of parents preferred using digital, social and newspaper meda for the awareness of early child care education policy while 73% (n=40) thought other channels can disseminate policy documents such as community channels, hospitals conducting propaganda and government. This indicates that the government should improve the implementation of policy documents in preschools and parents can learn the policies from the workshops conducted by preschools. Beside, social media such as the internet, public news and TV can spread the policies of ECCE to parents. Bennett and Neuman (2004) claim that the principal means of ensuring the quality of provision of policies is to provide intensive in-service training of teachers and parents. Therefore, it is necessary for the government to offer training opportunities for parents to learn and understand the policies of ECCE and know how to apply and follow the principles.

According to the findings from focus group interviews with 22 parents, themes that were derived are policy awareness and policy implementation as well as challenges of policy implementation. A total of 15 parents were not familiar with ECCE policies and 12 parents were unaware of the ECCE policies. This reveals that some parents lack awareness of policy in Malaysia. Parents proposed some issues of policy implementation in preschool from their perspectives such as poor hygiene and lack of direction by the welfare department in particular centres, and the situation of different policy implementation in preschools between rich and poor areas. However, the National Nutrition Policy, as well as the Child health Services Policy, were carried out to guarantee children’s health and safety ( *Early Childhood Care and Education Policy Implementation Review,* 2008). As the quality standard and assessment of policy implementation, the issue is that nobody checks the quality of ECCE centers from parents’ perceptions. Moreover, some parents point out there is the phenomenon of stressing children’s academic learning in particular preschool.

The key finding of the study is that stressing the importance of the government early child care education polices implementation should create awareness among the parents where most of the parents reported to have no idea about the government policy documents. Therefore, this study strongly recommends the government take on board the parents’ reports, and that polices regarding early child care eduation should engage the public via directly involving them or through media. The study finding is limited to the early child care education policy only and does not deal with high school or other educational subjects.

## Conclusions

Based on parents’ perceptions, ECCE policy is important. However, the majority of parents lack awareness towards the ECCE policies in Malaysia. The government of Malaysia should supply more information of ECCE policies to parents and focus on policy implementation. There are good practices of policy implementation from parents’ views. They expected quality early childhood care and education programs. The challenges of policy implementation of quality early childhood care and education programs remain. Without any doubt, the policy regarding parents’ awareness and implementation should be improved. There was the phenomenon of discrimination in some particular preschools. Also, there were gaps in quality in different preschools in the rich and poor areas. The government still faces problems as to how to standardise the policy implementation and assessment of the preschools’ practice. Therefore, the government should provide professional training for the teachers as well as provide training and learning opportunities to parents. Furthermore, the government should enforce the laws of the National Quality Assurance System and develop new policies that strongly support parent-preschool relationships to ensure the quality of preschool education.

In order to promote the quality of ECCE in public and private preschools, it is suggested that the Ministry of Education, the Ministry of Rural and Regional Development, as well as the National Unity Department and other related departments should enforce the implementation of the minimum standards and operational guidelines such as *National Preschool Quality Standard (SKPK).* The standards should outline essential components for preschools. National and District ECCE departments should be responsible for the integration of early childhood care and education and specific activities such as supervision of preschools. The government should provide professional training for the current and new teachers as well as provide training and learning opportunities to parents.

Besides that, the Ministry of Education and other educational departments should enforce the laws of the *National Quality Assurance System* and develop new policies that strongly support parent-preschool relationships to ensure the quality of preschool education such as the formulation of policy statements that clearly outline the roles of parents, teachers, and principals in children’s early education. Furthermore, the Quality Assurance Office should constantly assess the existing quality of ECCE of preschools in rural and urban areas in order to ensure that the licensed preschools meet the required minimum quality standards which should be developed and evaluated regularly to ascertain preschools owners adhere to and implement.

## Authors’ contributions

Dandan Tang; original draft and methodology, Fong Peng Chew; original draft and software, MohdNazri A.R; original draft and validation, Mogana Dhamotharan; Original draft, supervision, formal analysis. All authors have read and agreed to the published version of the manuscript.

## Data availability

### Underlying data

figshare: data spss11.22.sav. https://doi.org/10.6084/m9.figshare.19848607.v1 ( Abdul Rahman, 2022a)

This project contains the following file:

- data spss11.22.sav (raw SPSS data set)

Focus group data is not available without the relevant government department’s permission because this study is based on a government project. Because of this, and the detailed nature of the data, it is only available on request. To access the focus group transcript data, please contact the corresponding author mohdnazri_ar@um.edu.my. Any researcher affiliated with a university must show affiliation proof. Interview data is currently in Malay language and will be translated to English on request.

### Extended data

figshare: questionnaire.pdf. https://doi.org/10.6084/m9.figshare.19848604.v1 ( Abdul Rahman, 2022b)

This project contains the following file:

- questionnaire.pdf: (Questionnaire for the research and the focus group schedule)

figshare: excel spreadsheet SPSS Coding.xlsx. https://doi.org/10.6084/m9.figshare.20141057.v1 ( Abdul Rahman, 2022c)

This project contains the following file:

- excell spreadshet SPSS Coding (1).xlsx (data key for qualitative data file)

Data are available under the terms of the Creative Commons Attribution 4.0 International license (CC-BY 4.0).

## Author information

**Dandan Tang** is a final year doctorate student at the Faculty of Education, University of Malay (UM), Malaysia and about to submit her PhD Thesis. She is the author of a few research papers on the role of early child education and sustainable education polices published in Scopus and SSCI indexed journals.

**Fong Peng Chew** is a senior lecturer at Faculty of Education, University of Malaya (UM), Malaysia, teaching Malay language education and early childhood education program, and has presented approximately 160 working papers international and international seminars and conferences in Malaysia and foreign.

**Mohd Nazri Abdul Rahman** is senior lecturer at Faculty of Education, University of Malaya (UM), Malaysia. Teaching Malay language education and early childhood education, the author has been actively involved in the academic administrative duties with current active position Deputy of Dean and involved in more than 70 research projects on the education subjects, presented and attended multiple local and international conferences.

**Mogana Dhamotharan** is a full professor at the Faculty of Education, Segi University, Malaysia. She is the author of several research papers published in the Scopus and WOS indexed journals.
