# Supplementary material for: Parents’ perceptions on policies of early childhood care and education programmes in selected states of Malaysia
Source: F1000Res. 2022 Sep 29;11:938. Originally published 2022 Aug 16. [Version 2] doi: 10.12688/f1000research.122443.2 (PMC9535327; doi:10.12688/f1000research.122443.2)
Supplement: Peer review history

# Peer review: Reviewer response for version 2

**DOI:** 10.5256/f1000research.138551.r151970

**Published:** 2022-10-05

**Authors:** Abdul Halim Masnan

**Affiliations:** 1 Universiti Pendidikan Sultan Idris, Tanjung Malim, Malaysia

Approved 

 Thank you

Is the work clearly and accurately presented and does it cite the current literature?

Yes

If applicable, is the statistical analysis and its interpretation appropriate?

Yes

Are all the source data underlying the results available to ensure full reproducibility?

Yes

Is the study design appropriate and is the work technically sound?

Yes

Are the conclusions drawn adequately supported by the results?

Yes

Are sufficient details of methods and analysis provided to allow replication by others?

Yes

Reviewer Expertise:

Pedagogy in Early Childhood Education

I confirm that I have read this submission and believe that I have an appropriate level of expertise to confirm that it is of an acceptable scientific standard.

# Peer review: Reviewer response for version 1

**DOI:** 10.5256/f1000research.134430.r147732

**Published:** 2022-09-05

**Authors:** Hannah Ajayi, Johnson Okewole

**Affiliations:** 1 Obafemi Awolowo University, Ile-Ife, Nigeria; 2 Obafemi Awolowo University, Ife, Nigeria

The author(s) clearly pointed out the main problem of the study as the poor implementation of ECCE policies in Malaysia and lack of parental engagement. It is imperative to consider parents’ perception because it is a variable that can influence their engagement in the ECCE policies implementation and can boost the quality of ECCE. The author(s) therefore presented a succinct background on the policies on early childhood care and education in Malaysia in connections to the global agenda.

 However, the author(s) did not provide occasions that made them arrive at the conclusion that ‘the implementation is less than being satisfactory’. Is it that the quality is below standard? Who lacked ‘political will’? Parents or the government?

 To make the paper better, the author(s) can suggest the advantages of parents’ awareness of ECCE Policy. Of what benefits will it be to children, families and the nation at large. The author(s) should provide adequate information about the variables in Research Questions 2 and 3 in the Background – What are the ways that parents may know about the policies and of what benefits will it be if those ways are made available? What challenges surround quality ECCE that parents need to be aware of and what part can the parents play if they are aware?

 The mixed-methods adopted for the study is appropriate in that the focus group discussion helps to get an in-depth perception of parents beyond the surface level of using questionnaire only which may be subjective in nature. The results and findings were well presented, but the author(s) could take a step further to determine the factors that are responsible for a lack of awareness using the available data in the study. The findings are well presented. The study was well concluded however, the study’s limitation is that the experience in other countries have not been brought to bear. What obtains in other countries as regards parents' perception of ECCE policies implementation and quality may serve as future research.

Is the work clearly and accurately presented and does it cite the current literature?

Yes

If applicable, is the statistical analysis and its interpretation appropriate?

Yes

Are all the source data underlying the results available to ensure full reproducibility?

Yes

Is the study design appropriate and is the work technically sound?

Yes

Are the conclusions drawn adequately supported by the results?

Yes

Are sufficient details of methods and analysis provided to allow replication by others?

Yes

Reviewer Expertise:

Early Childhood Education

We confirm that we have read this submission and believe that we have an appropriate level of expertise to confirm that it is of an acceptable scientific standard.

# Peer review: Reviewer response for version 1

**DOI:** 10.5256/f1000research.134430.r147731

**Published:** 2022-08-18

**Authors:** Abdul Halim Masnan

**Affiliations:** 1 Universiti Pendidikan Sultan Idris, Tanjung Malim, Malaysia

After careful reading of the manuscript, I concluded that the paper has the potential to be 'Approved'. Authors have covered the Malaysian parents’ perception of government established policies for early child care education in the country. Parents' involvement in the educational policies is a remarkable step and it is parents’ right to know and understand what governments have planned and how schools are practically implementing them. This study is comprehensively drafted and well written, however, I have a few protentional but minor questions and directions for the authors to improve the paper more before it can be 'Approved':

**Review comments: **

1. Mention key contributions of the study in the ending section of the introduction.
2. Provide a reference for “ *The history of early childhood care and education started in the 1950s. From 1950 to 1960, kindergartens in Malaysia were opened by socialist Christians as part of church activities. Almost all kindergartens were owned by individual or private agencies in town areas*”. 
3. In my opinion, research aims and questions should be part of the introduction, however, it's also dependent on the publisher's policies if they do accept as a part of the literature review, authors are free to decide or refer to the publisher's policies. 
4. Section Method “ *Reflexivity*”, “ *did not face any problems e reaching out to the participants*” remove or rewrite the sentence.
5. Why Tadika schools are more than Taska? Table 1 shows the difference is very high – any reason please explain?
6. Results presented very well along with constructive discussion, however, if there is any study limitation, can explain it.  

Is the work clearly and accurately presented and does it cite the current literature?

Yes

If applicable, is the statistical analysis and its interpretation appropriate?

Yes

Are all the source data underlying the results available to ensure full reproducibility?

Yes

Is the study design appropriate and is the work technically sound?

Yes

Are the conclusions drawn adequately supported by the results?

Yes

Are sufficient details of methods and analysis provided to allow replication by others?

Yes

Reviewer Expertise:

Pedagogy in Early Childhood Education

I confirm that I have read this submission and believe that I have an appropriate level of expertise to confirm that it is of an acceptable scientific standard, however I have significant reservations, as outlined above.
